# Pre-season and in-season body composition assessment by bioimpedance in professional football athletes: implications for sports nutrition, physical performance, and hormonal health

**DOI:** 10.3389/fnut.2025.1657855

**Published:** 2025-08-01

**Authors:** Rand Iblasi, Mahmoud Abualsaud, Adam Tawfiq Amawi, Hadeel Ghazzawi

**Affiliations:** ^1^Department of Nutrition and Food Technology, School of Agriculture, The University of Jordan, Amman, Jordan; ^2^Department of Sport Sciences, Jordan Football Association, Amman, Jordan; ^3^Department of Movement Sciences and Sports Training, School of Sport Sciences, The University of Jordan, Amman, Jordan

**Keywords:** bioimpedance analysis, body composition, football players, seasonal variation, sports nutrition

## Abstract

Seasonal transitions in professional football specifically between the pre-season and in-season phases are accompanied by distinct physiological, hormonal, and nutritional demands. Understanding these fluctuations is essential to optimizing dietary periodization, improving performance outcomes, and supporting player recovery. This study aimed to assess and compare the dietary intake, hormonal biomarkers, and body composition of professional football players during the pre-season and in-season phases. A cross-sectional observational study was conducted on 15 professional male football players (mean age: 25.15 ± 3.78 years). Dietary intake was recorded over 7 consecutive days during each phase and analyzed using ESHA Food Processor software. Nutrient adequacy was evaluated against established sports nutrition guidelines. Physical performance (30-meter sprint, vertical jump, and Yo-Yo Intermittent Recovery Test Level 1), body composition (body weight, fat mass, and fat-free mass via bioimpedance), and hormonal biomarkers (GH, IGF-1, testosterone, insulin, cortisol) were also measured. Average daily energy and carbohydrate intake were higher during the in-season phase (3,240 kcal and 392.0 g, respectively) compared to pre-season (2,890 kcal and 349.6 g), though the differences were not statistically significant (*p* > 0.05). Protein intake was significantly higher during pre-season (168.79 ± 42.03 g vs. 140.86 ± 34.86 g, *p* = 0.02), whereas fat intake was significantly lower (98.26 ± 23.32 g vs. 131.04 ± 42.74 g, *p* = 0.01). Micronutrient analysis revealed significant phase-dependent differences in intake of vitamins B1, B2, B5, choline, calcium, sodium, and zinc (*p* < 0.05). Only GH levels showed a significant increase in-season (0.49 ng/mL vs. 0.19 ng/mL, *p* = 0.03); no other hormonal markers differed significantly. Despite increased physical demands, players failed to meet recommended energy and carbohydrate targets in both pre-season and in-season phases, while protein intake exceeded recommendations. Several micronutrient imbalances were also observed. These findings highlight the need for tailored, phase-specific nutritional strategies to support the health, hormonal balance, and performance of professional football players throughout the competitive season.

## Introduction

Football has become the most popular and widely played sport globally in recent decades (1); it is classified as an intermittent and high-intensity team sport, partly due to the high-intensity running, brief sprints, tackling and jumping activities that demand high energy, long recovery and may expose athletes to exhaustion (2). In recent years, the game is played faster and became more physical and aggressive demanding intense training and high physical fitness levels (3). In this context, nutrition plays a pivotal role in supporting athletic performance, recovery, and overall health, serving as a fundamental component of training and competition preparation (4).

Seasonal variation in football plays a critical role in shaping training strategies, performance outcomes, and recovery processes. Consequently, the maintenance of fitness during a season is one of the main targets of all football teams. The preparatory period focuses on rebuilding physical fitness and enhancing technical and tactical performance through high training volumes (5, 6). The competitive period involves frequent match play, requiring substantial energy expenditure but lower training intensity due to increased recovery needs (6). The transition period allows for physical and mental recovery through reduced or recreational activity, though prolonged inactivity may negatively impact physiological performance (5). These periods differ markedly in physiological demands, training intensity, and nutritional requirements, necessitating tailored approaches to support players’ adaptation, performance, and health across the competitive cycle.

Body composition is acknowledged as a determinant of athletic health and performance (7). It often improves in pre-season due to increased conditioning, while it remains more stable in-season as training shifts toward performance and recovery (8). Physical performance typically improves from pre-season to in-season but may stabilize or decline slightly due to fatigue and recovery needs (9). Besides, hormonal regulation plays a crucial role in athletic performance, recovery, and overall metabolic health (10). Hormonal levels in athletes can vary between pre-season and in-season due to changes in training intensity, volume, stress, and recovery (11). Moreover, little is known about the integrative analysis of individual physical performance and biochemical parameters during the season.

Greater attention must be directed toward optimizing the nutritional intake of football players in Jordan. To enhance performance, dietary strategies include maximizing the consumption of macronutrients and micronutrients and changing the composition of these foods and the timing of their consumption throughout the day (12, 13). Based on scientific evidence, international guidelines recommend the amounts, type, and timing of food intake to ensure excellent training while reducing the risk of injuries and illness (13). Therefore, there must be a proper balance between nutrition, training, and recovery to obtain metabolic optimization. Energy should be provided from various foods: carbohydrates, proteins, fat, and micronutrients. Energy balance maintenance is important for those who practice physical activity (14). It is generally acknowledged that balancing energy consumption and expenditure is essential to avoid an energy deficit or surplus. When establishing a dietary strategy, it is necessary to consider the shifting energy expenditure that occurs as a result of the training load to adjust the amount of energy used (15).

Maintaining adequate energy availability is essential for supporting athletes’ health, performance, and recovery throughout demanding training cycles (16). Moreover, when players are exposed to high volumes of training and (or) competition interspersed with insufficient recovery, could show signs of fatigue (17); which can contribute to the development of Relative Energy Deficiency in Sport (REDs). REDs arises from a mismatch between dietary energy intake and energy expenditure, leading to impaired physiological functions such as hormonal imbalance, decreased bone health, reduced endurance performance, and increased injury risk (18).

In high-performance sports, aligning nutritional strategies with the demands of training and competition is essential for optimizing performance and recovery (13, 15). Nutritional periodization, including the structured planning of weekly intake, is a key strategy in sports nutrition for optimizing athletic performance. It allows athletes to tailor their energy and nutrient consumption to the demands of individual training sessions and align dietary intake with specific weekly performance goals (19). This approach ensures that energy and macronutrient needs are met in accordance with training intensity and volume throughout the competitive season or training cycle. In football, various models of carbohydrate periodization have been proposed, which adapt intake according to the specific demands of each microcycle (20).

Understanding physiological and nutritional variations across different training phases is essential for optimizing athletic performance, preventing injuries, and supporting recovery (21). In professional football, such insight enables tailored interventions that align dietary intake and physical demands with the specific objectives of each season phase. Despite growing interest in training periodization and sports nutrition, limited research has comprehensively examined the integrated physiological and nutritional demands across the pre-season and in-season phases in professional football especially in the Middle East. Most studies focus either on performance metrics or isolated dietary factors, with few addressing how nutrition aligns with seasonal training loads. Additionally, there is a lack of data on individualized dietary strategies that adapt to the dynamic changes in workload, recovery, and performance goals throughout the competitive cycle (19, 21, 22). Therefore, this study aims to quantify and compare the nutritional, physical performance, and body composition changes across the pre-season and in-season phases in professional football players.

## Methods

### Participants

Fifteen professional footballers were monitored throughout the programmed pre-season and in-season periods. Players were chosen from Jordan National Football Team and collection of the sample was taken during pre-season in July. These players ranged in age from 19 to 35 (*n* = 15 age 25.15 ± 3.78 years, body mass 76.38 + 6.27 kg, height 179.85 + 6.53 m). This study took a place across one-week mid-season in the preparatory and in-season periods. During the study, two participants were excluded due to severe injuries, reducing the final sample size. The football players who participated in the study were a very homogeneous group characterized by a similar level of athletic performance, career duration, and applied training loads. Inclusion was limited to male participants, Jordanian nationality, exhibiting more than 5 yrs. of continued experience. In addition, players must participate in all training sessions across the two phases. Besides, players must keep a complete food diary for all days of the microcycle. Female athletes were not included due to physiological differences associated with the menstrual cycle, particularly fluctuations in cortisol and testosterone levels across different phases, which could potentially influence dietary intake and metabolism (23–25). Exclusion criteria included active smoking status including cigarettes, pipes, cigars, and e-cigarettes, known metabolic disease, cardiovascular disease, respiratory disorders, and orthopedic issues (within the past 5 years) limiting exercise performance. Additionally, any participant using anabolic steroids or currently taking medications (e.g., steroidal and non-steroidal) or dietary supplements (creatine, beta-alanine) that may interfere with the study results will be not enrolled in.

Participants were observed for 1 week of training during both the pre-season and in-season periods. The middle week of the pre-season training period was chosen for analysis as it was envisaged that this would provide the most representative micro-cycle to evaluate during this period. The in-season training analysis was carried out during week 24, when players were following a regular pattern of games and training. All players were notified of the research protocol, benefits and risks before providing written informed consent. In accordance with the approved research design authorized by the Faculty of Graduate Studies, the Deanship of Scientific Research, and the Institutional Review Board (IRB) at the University of Jordan (Decision Code: 250/2024), submitted by Prof. Hadeel Ali Ghazzawi from the School of Agriculture, written informed consent was obtained from each football player who participated in the study.

### Experimental design and study period

The football players participated in an observational study comprising measurements of energy intakes, dietary intakes, physical tests, hormons and body composition during pre-season and in-season. All data were collected during the 2024–2025 period, from July 2024 to June 2025. Data were gathered during both the pre-season and in-season periods.

**Figure 1:**
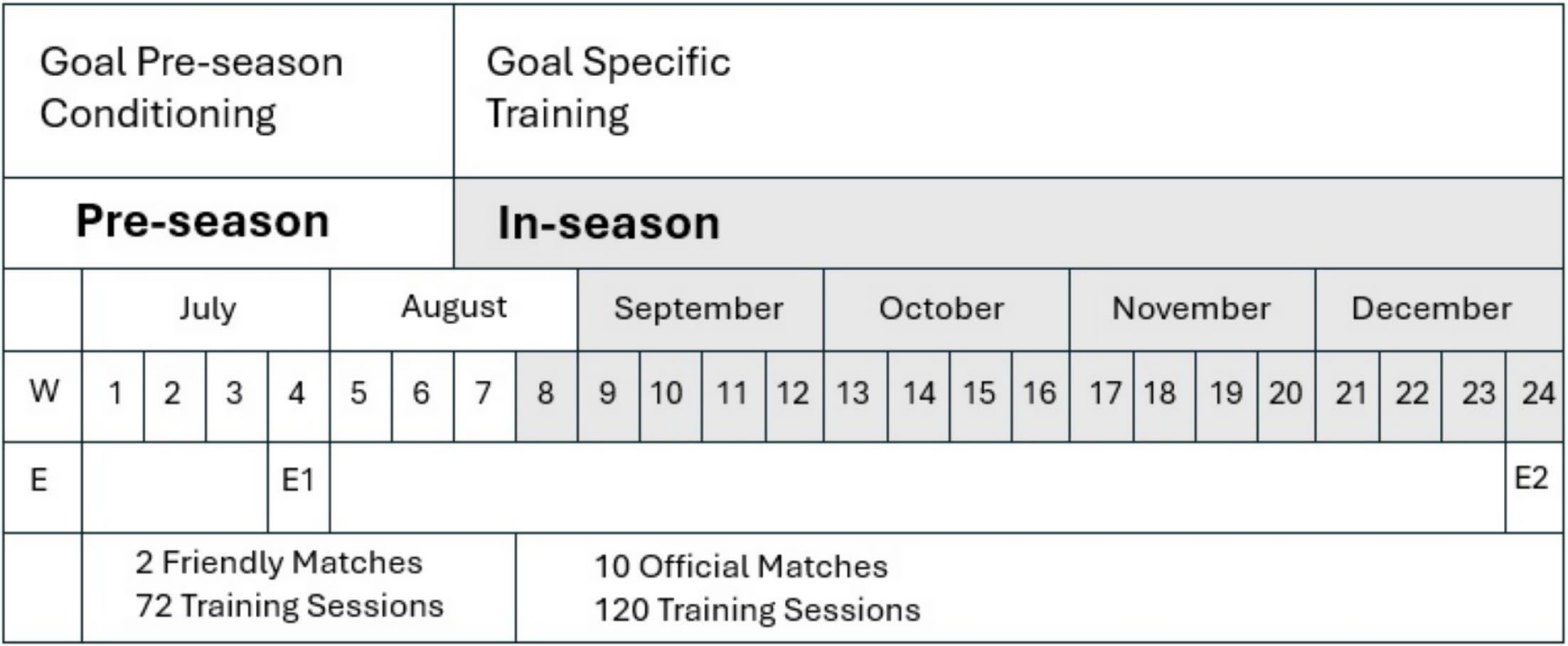
Seasons schedule. W, Week; E, Evaluation moment; E1, Evaluation moment 1 (mid of pre-season); E2, Evaluation moment 2 (mid of in-season).

The seasonal training structure of the Jordanian national football team was divided into a pre-season and an in-season phase (Figure 1). Prior to the structured training, an off-season (transition phase) was observed from the last 2 weeks of May until the end of June, providing players with adequate time for physical recovery and mental rejuvenation before the start of pre-season.

The pre-season, covering weeks 1 to 6 (beginning the first week of July and continuing through early August), included 2 friendly matches and 72 training sessions. Training occurred twice daily for 6 days per week, with each session lasting 2 h, totaling 4 h of training per day. The focus during this phase was on aerobic conditioning, muscular strength, and tactical preparation.

The in-season phase began in week 7 and extended through the second week of June, comprising 10 official matches and 120 training sessions. Training volume was reduced to once daily, with each session lasting 3 h, emphasizing match readiness and performance maintenance.

Two evaluation points were conducted: E1 in week 4 (mid pre-season) and E2 in week 24 (mid in-season), to monitor physical and performance adaptations across the season.

### Data collection

Data was collected from participants by interviewing them individually. The researcher conducted a face-to-face interview with each participant to collect all the necessary information. The researcher explained and filled out the demographic and health data personal questionnaire to each participant by asking questions and ensuring all participants understood and answered each question. Before commencing the study, seven dietary records were collected during subsequent visits or via phone. A survey was used to collect demographic and health-related data. The nutritional assessment consisted of four parts (food intake, health-related data, anthropometric measurements, and body composition).

### Dietary assessment

Participants were asked to provide a 7 day food record in pre-season and in-season. Telephone interviews, WhatsApp texts, and voice messages were conducted every day of recording to ensure the participant’s information was accurate and to obtain the details rapidly before participants failed to remember the data. The food dietary intake records were analyzed using (ESHA Food Processor SQL® software version 10.1.1; ESHA, Salem, Oregon State, United States) to energy, and essential nutrients were selected.

All data were collected in a single session at each time point, all visits took place in the same laboratory, the same equipment was used for all tests, and the data collection was performed by the same trained technician phases of Season. For data analysis, the season was divided into two distinct phases: pre-season (weeks 1–6) and in-season (weeks 7–42). Physical performance, body composition, and blood tests were conducted in pre-season week 4 and in-season week 24.

### Hormonal assessment

Blood tests were performed in an accredited laboratory. Participants visited the laboratory after 12 h of fasting, approximately 7 and 9 am, and were asked to abstain from caffeine, coffee, alcohol consumption, and vigorous physical activity for 12 h before each measurement. Fasting blood samples (8 mL) were obtained from participants under medical supervision after a 12-h fast. Blood tests, including Hormonal profiling (total testosterone, IGF-1, cortisol, insulin, and growth hormone levels), were estimated by standard enzymatic analysis. Participants were measured in pre-season week 4 and in-season week 24.

### Anthropometric measurements and body composition assessment

Weight was measured for participants before training at the lowest level of clothing, with accuracy to the nearest 0.1 kg, using a digital scale by Bioelectrical Impedance Analysis (Inbody Co., Ltd., Seoul, KOREA). Participants were asked to stand barefoot on the scale. Height was measured using a stadiometer (ADE MZ10023-1, Telescopic height measure for scale and wall mounting). When height was measured, participants were barefoot and standing in a relaxed position, with arms hanging freely (to the nearest 0.5 cm) with an accuracy of 1 mm. Bioelectrical Impedance Analysis measured fat and lean mass. Participants were measured in pre-season week 4 and in-season week 24.

### Training program assessment

During pre-season training period, participants trained 6 days a week, including friendly matches. The physical training programs included mainly aerobic and mixed aerobic-anaerobic type activities with and without the ball as well as speed, strength, stretching and coordination training. In addition, training involved technical drills and team tactics (Figure 1).

Training sessions were mainly devoted to technical-tactical skill development. The seasonal schedule and evaluation points are illustrated in Figure 1.

Both sets of tests were performed using the same procedures, at the same time of the day, under the same environmental conditions, and by the same examiner. All measurements were taken at the same time of the day. During the 24 h before each test, no intensive training was allowed. Players maintained their training program (intensity, duration, and frequency) as before the experiment and training periodization was implemented by the club’s coaching staff. Coaches, strength and conditioning professionals, and the medical staff were the same during both seasons. Over the course of the study, all players were already familiar with the testing procedures as it is part of their usual fitness assessment program.

Participants performed specific tests, including endurance, speed, and strength, based on the team physical trainer’s requests (26). The three tests were the Yo-Yo intermittent recovery test in its level 1 version (i.e., Yo-Yo IR1), 30 m sprint, and vertical jump tests. All evaluation sessions were performed at the same time of the day between 1.00 p.m. and 3.00 p.m. In this regard, the Yo-Yo IR1 was reported as a relevant measure of mainly aerobic intermittent high-intensity endurance in football (i.e., criterion-convergent validity). Furthermore, the Yo-Yo IR1 was shown to be related to match activities performed at high intensity assumed as a key variable in competitive football. Given this, the Yo-Yo IR1 may be used to estimate players’ capability to perform at a high intensity during the game (27). The Yo-Yo test was performed according to the procedures suggested by Castagna et al. (28). The test consists of 20-m shuttle runs performed at increasing velocities with 10 s of active recovery between runs until exhaustion. Audio cues of the Yo-Yo test were recorded on a CD^1^ and broadcasted using a portable calibrated CD player (Philips, Az1030 CD player, Eindhoven, Holland). Before the test, all subjects carried out a warm-up period consisting of the first four running bouts in the test (29). The total test duration was 6–20 min (30). The end of the test is considered when the participant twice fails to reach the front line in time (i.e., objective evaluation) or feels unable to complete another shuttle at the dictated speed (i.e., subjective evaluation) (29). The total distance (TD) covered during the Yo-Yo test level-1 (including the last incomplete shuttle) was calculated and stored for further analysis (31). Furthermore, the estimated VO₂ max was calculated using the Yo-Yo intermittent recovery test, following the equation: VO₂ max (mL·kg^−1^·min^−1^) = (distance covered in meters × 0.0084) + 36.4 (27).

In the second test, the 30-meter sprint test, running speed is a parameter that determines the body’s potential to run over a specific distance in the shortest time possible. The test requires a flat, non-slip surface, a stopwatch, and an assistant. The athlete warms up for 10 min, and then the assistant marks out a 30-meter straight section with a cone. Participants began the sprint at a self-selected time and performed a maximal-effort run over a 30-meter distance. The assistant starts the stopwatch on the athlete’s first foot strike after starting and stops the stopwatch as the athlete’s torso crosses the finish line. Sprint time was recorded in seconds using a stopwatch. Participants were allowed two trials for each sprinting distance, and the best time was used for analysis (32).

The third test is the vertical jump, specifically the countermovement jump (CMJ), which assesses anaerobic performance and is considered a key indicator of lower-body explosive strength in professional football players. It included four data collection trials and was performed using the Optojump Next (Microgate, Bolzano, Italy) system of analysis and measurement. Before the initiation of the measurements, a 10-min warming up was applied. In this test, participants were encouraged to jump to maximum height, and they began in an erect standing position and moved into a semi-squat position before jumping. Before data collection, each participant performed three experimental trials to ensure correct execution. In the CMJ protocol, participants began in a tall standing position, with feet placed hip-width to shoulder-width apart. Then, participants dropped into the CMJ position to a self-selected depth, followed by a maximal-effort vertical jump. Participants were instructed to keep their hands on their hips throughout the entire movement to eliminate the influence of arm swing (33). A trial was repeated if the participant removed their hands from the hips at any point or exhibited excessive knee flexion during the CMJ. The participants reset to the starting position after each jump.

Dietary intake, hormone levels, and body composition were measured during pre-season week 4 and in-season week 24, while physical performance tests were conducted in the same weeks but in on different days.

### Statistical analysis

A paired *t*-test was conducted to compare pre-and post-intervention outcomes, including hormonal responses, body composition, and physical performance parameters. All data was collected and entered into the Statistical Program for Social Studies (SPSS17.0.1.2008, Chicago: SPSS Inc.) software. All data are presented as mean± SD and the level of statistical significance was set at (*p* ≤ 0.05). A seven-day average was calculated for total energy intake (kcal), macronutrients (expressed as % of total energy intake, grams, and grams per kilogram per day), and micronutrients.

## Results

### Dietary intake

Table 1 shows the significant differences between pre-season and in-season were observed in dietary protein intake (g) (*p* = 0.02) and dietary fat intake (g) (*p* = 0.01). Other nutrient intakes showed no statistically significant changes.

**Table 1:** Dietary intake of elite football players during pre-season and in-season periods.

Nutrient	Dietary intake (M ± SD)		RV	*p*-value
	Pre-season (P)	In-season (IN)		
Average intake	2889.87 ± 815.33	3239.88 ± 792.78	–	0.14
Energy (kcal/kg)	38.24 ± 11.71	41.76 ± 11.33	40–70^1^	–
CHO (g)	349.55 ± 138.13	392.01 ± 123.29	–	0.27
CHO (g/kg/day)	4.66 ± 2.02	5.08 ± 1.75	(P) 4–8 g/kg^2^ (IN) 3–8 g/kg^2^	
CHO (%)	48%	48%	50–60^3^	
Dietary Prot (g)	168.79 ± 42.03	140.86 ± 34.86	–	0.02*
Dietary Prot (g/kg/day)	2.21 ± 0.60	1.82 ± 0.52	1.2–2^4^	–
Dietary Prot (%)	23%	17%	15–20	–
Dietary Fat (g)	98.26 ± 23.32	131.04 ± 42.74	–	0.01*
Dietary Fat (g/kg/day)	1.29 ± 0.29	1.69 ± 0.56	–	–
Dietary Fat (%)	29%	35%	25–35^5^	–

RV, Recommended Values (RV) are adapted from international sports nutrition guidelines, including the International Society of Sports Nutrition (ISSN), the International Olympic Committee (IOC), the American College of Sports Medicine (ACSM), and the UEFA Expert Group Statement on Nutrition in Elite Football. See references 1: (49, 69). 2: (13). 3: (13, 70, 71). 4: (35, 70). 5: (49). SD, Standard deviation. *p < 0.05, statistically significant.

Table 2 shows significant differences in micronutrient intake between pre-season and in-season phases. Intakes of vitamin B1 (*p* = 0.02), vitamin B2 (*p* = 0.02), vitamin B5 (*p* = 0.04), choline (*p* < 0.001), calcium (*p* = 0.03), sodium (*p* = 0.04), and zinc (*p* = 0.02) differed significantly between the two periods. Other micronutrients did not show statistically significant changes.

**Table 2:** Micronutrients intake pre- and in-season compared to recommended values.

Micronutrients intake	Mean intake Pre-season ±SD	Mean intake In-season ±SD	EAR-UL (HC DRI)	*p*-value between
Vit A (μg)	1138.55 ± 3160.20	343.81 ± 116.84	625–3000^2^	0.37
B1 (mg)	1.98 ± 0.87	5.35 ± 4.76	1.2**^2^	0.02*
B2 (mg)	2.32 ± 1.69	5.72 ± 5.18	1.3**^2^	0.02*
Niacin eq (mg)	38.67 ± 16.80	35.26 ± 6.91	12–35	0.42
B5 (mg)	10.51 ± 8.11	5.87 ± 1.38	5**	0.04*
B6 (mg)	3.49 ± 2.99	1.94 ± 0.44	1.1–100	0.07
B12 (μg)	12.55 ± 24.28	3.48 ± 1.54	2–2.4	0.19
Biotin (μg)	22.14 ± 10.36	24.95 ± 10.57	30**	0.35
Vit C (mg)	166.10 ± 124.90	146.64 ± 64.48	75–2000	0.53
Vit D (mg)	1.12 ± 0.02	1.32 ± 1.15	10–100	0.54
Vit E (mg)	26.81 ± 73.90	7.61 ± 1.99	12–1,000	0.36
Folate (μg)	533.84 ± 199.39	519.31 ± 117.54	320–1,000	0.77
Vit K (μg)	80.89 ± 72.38	51.32 ± 15.86	120**	0.13
Choline (mg)	253.09 ± 212.07	23.50 ± 128.98	550–3,500**	0.00*
Calcium (mg)	843.68 ± 501.63	549.90 ± 190.20	800–2,500	0.03*
Cupper (mg)	2.59 ± 4.84	1.17 ± 0.31	0.7–10	0.30
Iodine (μg)	79.17 ± 44.98	105.12 ± 56.83	95–1,100	0.09
Iron (mg)	20.35 ± 7.98	17.66 ± 2.26	6–45	0.20
Magnesium (mg)	367.05 ± 176.81	276.59 ± 61.23	330–350	0.06
Manganese (mg)	4.43 ± 2.75	3.90 ± 0.10	2.3–11**	0.49
Phosphorus (mg)	1035.25 ± 522.47	1008.12 ± 229.22	580–4,000	0.83
Potassium (mg)	2907.17 ± 756.91	2962.09 ± 762.55	3400**	0.80
Selenium (μg)	105.23 ± 59.83	89.41 ± 31.00	45–400	0.29
Sodium (mg)	3173.78 ± 733.99	2698.40 ± 714.99	1,500–2,300**	0.04*
Zinc (mg)	19.12 ± 14.93	9.25 ± 2.25	9.4–40	0.02*

EAR, Estimated average requirement; UL, Tolerable upper intake level; HC DRI, Health Canada’s Dietary Reference Intakes Tables for Elements (2020). **not determined. *p < 0.05, statistically significant. Source: [2]. Available at: https://www.canada.ca/en/health-canada/services/food-nutrition/healthy-eating/dietary-reference-intakes/tables/reference-values-elements.html.

Table 3 shows that among the measured variables, only growth hormone (GH) levels differed significantly between pre-season and in-season periods (*p* = 0.03). No significant changes were observed in body composition, physical performance tests, or other hormonal parameters.

**Table 3:** The average and standard deviation of variables in pre- and in-season compared to recommended values.

Variables	Pre-season	In-season	*p*-value
Body composition			
Weight (kg)	76.38 ± 6.27	77.58 ± 6.79	0.52
Fat (kg)	9.58 ± 2.23	9.67 ± 2.20	0.89
Fat %	12.48 ± 2.78	12.42 ± 2.52	0.93
Muscle mass (kg)	38.48 ± 3.24	39.04 ± 3.54	0.56
Muscle mass (%)	50.37 ± 5.92	50.33 ± 6.34	0.98
Physical tests			
30 m speed (s)	4.32 ± 0.21	4.27 ± 0.17	0.37
Vertical jump (cm)	45.75 ± 5.97	48.15 ± 6.08	0.18
Yo-Yo level 1(m)	1618.46 ± 409.75	1843.08 ± 722.67	0.22
Estimated VO₂max (mL·kg^−1^·min^−1^)	49.95 ± 3.39	51.88 ± 6.06	0.21
Hormonal parameters			
GH (ng/mL)	0.19 ± 0.22	0.49 ± 0.51	0.03*
Insulin (pmol/L)	65.38 ± 27.95	52.91 ± 24.02	0.11
IGF (ng/mL)	218.13 ± 46.68	223.54 ± 49.15	0.69
Total Testosterone (nmol/L)	25.05 ± 4.90	25.08 ± 6.62	0.98
Cortisol (nmol/L)	424.67 ± 131.20	405.78 ± 87.05	0.57

## Discussion

This study examined seasonal changes in dietary intake, physical performance, body composition, and hormonal biomarkers among players of the Jordan national football team. The results revealed insufficient energy and carbohydrate intake across both pre-season and in-season periods, while protein intake exceeded the recommended level during the pre-season (2.21 ± 0.60 g/kg/day), and fat intake remained within the recommended range (25–35% of total energy) in both phases, reaching the upper limit during the in-season. Several micronutrients, including vitamin D, vitamin K, choline, and potassium, were consistently below recommended thresholds, with sodium intake exceeding the upper limit. Only growth hormone levels increased significantly in-season, while other variables remained stable. These findings highlight the need for individualized nutrition strategies to support performance, recovery, and overall health throughout the season.

To the best of our knowledge, this is the first study conducted in Jordan to investigate seasonal variations in dietary intake, physical performance, body composition, and hormonal biomarkers among elite football players. While limited research of this nature exists in the broader Middle East region (34), comprehensive studies integrating all these variables remain scarce. This study fills a critical gap and offers valuable region-specific insights to support evidence-based nutrition and training strategies for football players.

Pre-season energy intake (38.24 ± 11.71 kcal/kg/day) was slightly below the lower end of the recommended range (40–70 kcal/kg/day), while in-season intake (41.76 ± 11.33 kcal/kg/day) just met the minimum threshold. This suggests that players may have been close to an energy deficit during pre-season, which can impair performance and recovery. Although carbohydrate intake accounted for ~48% of total energy in both phases, this falls slightly below the recommended https://www.canada.ca/en/health-canada/services/food-nutrition/healthy-eating/dietary-reference-intakes/tables/reference-values-elements.html50–60% range and may indicate a relative insufficiency given the players’ physical demands. Statistically significant differences between pre-season and in-season were observed for protein intake, which decreased from 168.79 ± 42.03 g to 140.86 ± 34.86 g (*p* = 0.02), and fat intake, which increased from 98.26 ± 23.32 g to 131.04 ± 42.74 g (*p* = 0.01). However, overall macronutrient intake was not significantly different between phases. These findings suggest that players’ macronutrient consumption and total energy intake remain relatively stable throughout the week, indicating a lack of adjustment in dietary intake to match variations in training intensity. This aligns with previous research in professional Australian Football athletes, where energy intake and carbohydrate-derived energy did not vary according to training day (35).

Multiple studies have assessed energy intake in football players during pre-season and in-season phases, revealing some variability across populations and competitive levels. In the pre-season, energy intake typically ranges from approximately 2,246 to 3,456 kcal/day, as reported in studies by Devlin et al. (36); Lee et al. (37, 38), and Książek et al. (39). In alignment with these findings, the current results demonstrated an average energy intake of 2,890 ± 815.33 kcal/day during the pre-season phase, consistent with values reported among collegiate and professional football players internationally. During the in-season, energy intake tends to increase modestly to support competition demands, with values around 2,800 to 3,400 kcal/day reported by Randell et al. (40) and Anderson et al. (21). These findings correspond with the present study, in which an average intake of 3,240 ± 792.78 kcal/day was recorded. Although energy intake in several studies generally meets estimated requirements, values below recommended levels have been reported in investigations by Devlin et al. (36), Lee et al. (37, 38), and (39). Such variations are likely attributable to differences in training intensity, match frequency, and individual player characteristics.

Therefore, periodized nutritional strategies are recommended to better align energy intake with training demands, ensuring a slight positive energy balance that optimizes performance and supports growth and development (41). Conversely, a negative energy balance characterized by an energy deficit occurring when energy expenditure exceeds intake coinciding with heavy training over a sustained period may cause detrimental effects on health. This imbalance can impair optimal development, as well as lead to performance decrements and an increased risk of injury (42). These outcomes are consistent with the clinical manifestations of Relative Energy Deficiency in Sport (REDs). Identifying and addressing negative energy balance is therefore critical for maintaining athlete health and optimizing performance throughout the season (43).

In accordance with established recommendations outlined by UEFA (13) and by Williams and Rollo (44), football players should consume 4–8 g/kg of CHO during pre-season to meet high training demands. During the in-season, intake should range from 3–8 g/kg with one game per week, and increase to 6–8 g/kg during congested fixture periods to support recovery and performance. Although carbohydrate intake was within the general recommended range of 3–8 g/kg/day, the observed values were at the lower end. Considering the players’ training load and recovery needs, these levels may not fully support optimal glycogen resynthesis. UEFA guidelines recommend 6–8 g/kg/day during congested fixtures, underscoring the potential inadequacy in intake. However, values were at the lower end of these ranges, which may be suboptimal for supporting maximal training adaptations and recovery. This approach may be detrimental to performance during training and competition. Evidence suggests that commencing exercise with suboptimal glycogen stores impairs physical output; for instance, Saltin (1973) observed that football players with reduced muscle glycogen ran shorter distances and exhibited diminished running intensity, particularly during the second half of matches. Accordingly, it is recommended that athletes increase dietary carbohydrate intake to ensure adequate glycogen availability, thereby supporting optimal performance during competitive play. Football specific studies have identified optimal carbohydrate intake to improve total match distance and ability to perform at high-intensity (44). High-carbohydrate diets are known to enhance muscle glycogen stores, which play a critical role in delaying fatigue and sustaining performance, particularly during prolonged or high-intensity exercise (19, 45).

Studies examining carbohydrate intake in football players report similar patterns across pre-season and in-season phases. During the pre-season, carbohydrate consumption typically ranges between approximately 4.6 and 5.4 g/kg/day, as reported by Książek et al. (39); Conejos et al. (46); and Raizel et al. (2) which closely aligns with the intake observed in the current study (4.66 ± 2.02 g/kg/day). In the in-season, intake generally increases slightly, ranging from about 4.7 to 6.4 g/kg/day Anderson et al. (21); Brinkmans et al. (47); Bettonviel et al. (48), which is consistent with the value observed in this study 5.08 ± 1.75 g/kg/day. Despite remaining within the broadly recommended range of 3–8 g/kg/day, these intakes tend to cluster at the lower end, which may not be optimal during periods of intensified training or congested fixtures.

Current recommendations for football players suggest a protein intake of approximately 1.2–2.0 g/kg/day to support muscle repair and adaptation (ISSN/IOC), while fat should contribute 25–35% of total energy intake to maintain essential physiological functions (49). In the present study, protein intake was significantly higher in the pre-season (168.79 ± 42.03 g) compared to the in-season (140.86 ± 34.86 g; *p* = 0.02), whereas fat intake was significantly lower during the pre-season (98.26 ± 23.32 g) compared to the in-season (131.04 ± 42.74 g; *p* = 0.01). These results align with previous research showing that young athletes often meet or exceed protein recommendations, even when in a state of negative energy balance (20). While optimal protein intake is essential for providing the amino acids necessary for the development of lean body mass (50), caution is warranted. In the context of insufficient energy availability, protein may be increasingly utilized as an energy source, thereby compromising its role in muscle protein synthesis and recovery (20).

Significant differences in micronutrient intake were identified between the pre-season and in-season periods, specifically for vitamin B1 (*p* = 0.02), vitamin B2 (*p* = 0.02), vitamin B5 (*p* = 0.04), choline (*p* < 0.001), calcium (*p* = 0.03), sodium (*p* = 0.04), and zinc (*p* = 0.02). In contrast, the intake of other assessed micronutrients did not exhibit statistically significant variation between the two phases, suggesting that their consumption remained relatively stable across the pre-season and in-season periods. However, intakes of vitamins B1 (thiamin), B2 (riboflavin), B5 (pantothenic acid), calcium, and zinc were significantly higher during the pre-season phase. Notably, sodium intake during the in-season period (2698.40 ± 714.99 mg/day) exceeded the general upper intake level (UL) of 2,300 mg/day set for the general population. However, this reference value may not fully apply to athletes, as sodium requirements vary depending on individual sweat rate, environmental conditions, and training intensity (51). Similar study results in terms of increased sodium intake were found in those training in judo (52), CrossFit (53), and soccer (2, 39, 51). According to Coles and Luetkemeier (54), excessive dietary sodium consumption among athletes is a common occurrence, often attributed to the frequent intake of processed foods particularly items such as processed turkey products and the regular consumption of fast food, especially after matches.

Vitamin D, vitamin K, and choline intakes were found to be below the Estimated Average Requirement (EAR) in both the pre-season and in-season phases, indicating a potential risk of inadequate intake among the athletes. These nutrients play critical roles in bone health, immune function, blood clotting, and cellular metabolism factors that are particularly important for athletic performance and recovery (55, 56). Furthermore, potassium intake was below the Adequate Intake (AI) level in phases, suggesting suboptimal consumption of this essential electrolyte, which is vital for maintaining fluid balance, nerve function, and muscle contraction (51, 57, 58). Persistent inadequacy in these micronutrients may compromise physiological function, increase the risk of injury, and impair recovery and performance in high level football players.

The examination of hormonal parameters revealed a significant increase in growth hormone (GH) concentrations during the in-season compared to the pre-season (*p* = 0.03), suggesting an adaptive endocrine response to the demands of competitive play. No significant changes were observed in insulin, insulin-like growth factor-1 (IGF-1), total testosterone, or cortisol levels between the two phases (*p* > 0.05). These results are consistent with previous research indicating that GH may fluctuate in response to training intensity and physical stress, while other hormones such as testosterone and cortisol tend to remain more stable in well-conditioned athletes (59, 60) The stable levels of anabolic and catabolic hormones may reflect effective recovery and balanced training loads throughout the season, which is critical for maintaining performance and reducing the risk of overtraining.

The body composition is a very important aspect to the physical ability level of the professional athletes in any modality, as the fat surplus can substantially decrease the human performance (34). The findings indicated that body weight, fat mass, body fat percentage, and muscle mass did not differ significantly between the pre-season and in-season phases (*p* > 0.05), suggesting overall stability in body composition across the two periods. These findings align with previous research demonstrating minimal seasonal variation in body composition among elite football players when training and dietary practices are appropriately managed (34, 61). The maintenance of stable body composition is essential for sustaining performance and reducing injury risk throughout the competitive season. However, contrasting findings have been reported in other studies. For example, McEwan et al. (62) observed significant reductions in fat mass and increases in lean mass during the pre-season period. Similarly, Milanese et al. (63) documented comparable improvements in body composition specifically, decreased fat mass and increased fat-free mass across the competitive season.

The physical performance measures, including 30 m sprint time, CMJ, Yo-Yo intermittent recovery test distance, and estimated VO₂ max, showed no statistically significant differences between the pre-season and in-season periods (*p* > 0.05), indicating relative stability in athletic performance throughout the competitive cycle. These results are consistent with previous studies that have reported minimal seasonal variation in speed, power, and aerobic capacity among elite football players when training and recovery are adequately managed. Consistent with the findings Fessi et al. (34) which conclude that both 30-m sprint and CMJ performances remained stable, with no significant differences observed (*p* = 0.99 and *p* = 0.34, respectively). Similarly, Lago-Peñas et al. (64) showed no differences were observed in CMJ and VO₂ max between the 2 phases.

In contrast, other studies have reported improvements in specific fitness parameters across seasonal phases. Clark et al. (65) observed a significant enhancement in CMJ performance between pre-season and in-season (*p* = 0.03), although VO₂ max remained unchanged. Similarly, Meckel et al. (66) reported significant improvements in vertical jump performance from pre-season (37.0 ± 5.3 cm) to mid-season (40.3 ± 5.5 cm; *p* < 0.05). Magal et al. (67) demonstrated a significant increase in VO₂ max (51.05 ± 5.97 vs. 54.64 ± 4.90 mL/kg/min) along with reduced sprint times over 10 m (2.03 ± 0.15 vs. 1.96 ± 0.11 s) and 30 m (4.72 ± 0.26 vs. 4.51 ± 0.24 s). Castagna et al. (68) also reported a significant improvement in Yo-Yo IR1 performance during the pre-season (*p* = 0.001), increasing from 2000 ± 279 m to 2,390 ± 409 m, along with a notable rise in VO₂ max post-training (*p* < 0.01). Likewise, Eliakim et al. (66) found significant improvements in VO₂ max and sprint times, while vertical jump performance remained unchanged during the pre-season phase. Discrepancies between studies may be explained by differences in training periodization, competition level, and player conditioning, highlighting the need for individualized monitoring to optimize performance adaptations. Although no statistically significant changes were observed, the inclusion of aerobic and anaerobic performance indicators (e.g., VO₂max, sprint time, and vertical jump) provides valuable insight into the athletes’ physiological status and adaptations across training phases.

In conclusion, the present study demonstrated that elite Jordanian football players maintained stable body composition and physical performance characteristics across the pre-season and in-season periods. Significant seasonal variation was only observed in growth hormone levels, reflecting an endocrine adaptation to competitive demands, while other hormonal markers remained unchanged. In addition, the dietary intake showed imbalances, with insufficient energy, carbohydrate, and micronutrient intake, alongside excessive protein and adequate to slightly excessive fat intake, highlighting the need for targeted nutritional monitoring throughout the season. These findings underscore the importance of carefully managed training, nutrition, and recovery strategies to sustain optimal physiological status and performance, while minimizing injury risk. Future research should explore individualized interventions to further enhance seasonal adaptations in this population.

This study has several limitations that should be acknowledged. The relatively small sample size may limit the generalizability of the findings to a broader population of football players. Additionally, the study’s observational design restricts the ability to establish causal relationships between training phases and physiological or nutritional changes. Dietary intake was self-reported, which may introduce reporting bias or inaccuracies. However, the study’s strengths include its longitudinal design, assessing multiple relevant parameters including body composition, physical performance, hormonal profiles, and micronutrient intake across distinct training phases within a well-defined elite athlete cohort. Furthermore, this research represents one of the first investigations of its kind in Jordan, providing valuable baseline data for this population and contributing to a better understanding of seasonal adaptations in Middle Eastern football players.

## Data Availability

The original contributions presented in the study are included in the article/supplementary material, further inquiries can be directed to the corresponding author/s.

## Footnotes

^1^www.theyoyotest.com, Ancona, Italy.
